# Health insurance type, healthcare utilization and out-of-pocket expenditure in the face of COVID-19: Evidence from Thai national survey data

**DOI:** 10.1371/journal.pone.0321468

**Published:** 2025-04-08

**Authors:** Chantal Herberholz, Piraya Saichol, Kannika Damrongplasit

**Affiliations:** 1 Centre of Excellence for Health Economics, Faculty of Economics, Chulalongkorn University, Bangkok, Thailand; 2 Thai Health Information Standards Development Centre, Nonthaburi, Thailand; University of Nigeria, Enugu Campus, NIGERIA

## Abstract

**Objectives:**

Universal population coverage for healthcare was achieved in several countries, including Thailand, while retaining fragmented health insurance schemes. Fragmentation in health financing has been debated since it can exacerbate inequalities, especially when health systems are under stress due to a public health emergency. This study examines whether the type of public health insurance affects outpatient healthcare utilization and out-of-pocket expenditure in Thailand before and during the coronavirus pandemic.

**Methods:**

Using the 2019 and 2021 waves of the nationally representative Health and Welfare Survey and a repeated cross-sectional design, logit and multinomial logit models are estimated to investigate the effect of health insurance type on outpatient healthcare utilization (n=10,220), while two-part and Tobit models are employed as alternative models for the analysis of out-of-pocket expenditure (n=12,014). For both healthcare utilization and out-of-pocket expenditure, the study also explores models with and without interactive terms between insurance coverage type and a dummy variable capturing the COVID-19 period.

**Results:**

Type of health insurance is found to impact provider choice (i.e., designated versus non-designated providers) rather than outpatient care utilization per se. Insignificant interaction effects indicate further that the relationship between health insurance type and outpatient care utilization is not affected by the pandemic. The regression results also show that health insurance type is associated with out-of-pocket expenditure (separated into medical and transportation spending) but the magnitude of the effect is relatively small, pre- and peri-pandemic. High-need persons with, for example, chronic conditions, however, face a higher out-of-pocket burden in terms of medical and transportation spending.

**Conclusion:**

Overall, the results suggest that Thailand’s universal health coverage system has continued to live up to its promise of access and financial protection in the face of COVID-19, despite existing fragmentation. Notwithstanding, this study highlights that universal health coverage is an ongoing effort that requires careful monitoring, inter alia to mitigate undesirable consequences of fragmentation and to ensure that high-need and other vulnerable persons are not left behind.

## Background

Several countries have maintained specific health insurance schemes for certain population groups such as civil servants and formal sector employees when advancing towards universal health coverage. This fragmentation can exacerbate existing inequalities across population segments [1].

Thailand is among the countries with specific health insurance schemes for defined population groups. Universal population coverage was achieved in 2002 when the third scheme, the Universal Coverage Scheme (UCS), was implemented for those neither covered by the other main schemes, that is the Civil Servant Medical Benefit Scheme (CSMBS) and the Social Security Scheme (SSS), nor any other public scheme. Although the three main schemes all offer a comprehensive health benefit package, scheme characteristics differ (Table A1 in S1 Appendix).

The UCS has been acclaimed nationally and internationally as a successful, pro-poor universal health coverage reform, accentuating that universal health coverage is a critical component of the United Nations’ Sustainable Development Goals. Healthcare utilization increased for those covered by the UCS, most notably the previously uninsured [2,3] and former beneficiaries of the medical welfare scheme for the poor [4]. The benefit incidence analysis by Limwattananon et al. [5] further showed that poorer wealth quintiles benefitted to a larger extent from public subsidies for outpatient and inpatient services than richer wealth quintiles given their higher utilization of healthcare services and low out-of-pocket health expenditure (OOP). Other research articles demonstrate that the incidence of catastrophic health expenditure and impoverishment due to OOP substantially decreased after the introduction of the UCS [6,7]. Catastrophic and impoverishing health spending was mainly caused by using services not covered by the UCS benefit package and bypassing designated providers [6]. The benefit package of the UCS has been expanded since its inception and only very few services are excluded [8]. Renal replacement therapy, for example, was included in the benefit package of the UCS in 2008 [9]. As a result, all three public health insurance schemes offer a comprehensive benefit package although the benefits of the CSMBS tend to be more generous (Table A1 in S1 Appendix). More affluent groups may opt for non-designated providers for reasons of convenience and better perceived quality of care [7].

In Thailand, OOP as a percentage of total health expenditure decreased from 34.19% in 2000 (i.e., pre-UCS) to 8.67% in 2019 [10]. Using data from the 2000 and 2004 waves of the Socio-Economic Survey (SES), the analysis by Limwattananon et al. [11] highlighted that monthly household OOP of the UCS target population decreased by 28% on average, with the effect being most pronounced in case of outpatient care. Nearly all studies that examine OOP using Thai data focus on the UCS reform and conclude that it assures financial protection. There are only two studies that examine the determinants of OOP using Thai data [12,13]. Both studies focus on the pre-UCS reform period and do not control for health insurance status.

In a recent study, using 1996–2015 SES data, Tangcharoensathien et al. [14] confirmed the UCS’ long-term success in terms of reducing catastrophic and impoverishing health spending. Their dataset, however, does not include the coronavirus pandemic years. The pandemic of the coronavirus disease 2019 (COVID-19) affected healthcare utilization and OOP around the world. Yet, the direction of the effects is ambiguous [15–18]. Moynihan et al. [17] concluded that healthcare utilization decreased by about one third during the COVID-19 pandemic in the predominantly high-income countries covered by their systematic review. Greater disruptions in the delivery and utilization of essential healthcare services caused by various demand and supply factors were found in low- and middle-income countries than in high-income countries [19]. In three out of five countries (i.e., in Belarus, Mexico, Peru, but not in Russia and Viet Nam), health systems failed to provide protection against catastrophic health expenditures or maintain access to healthcare services in 2020 [15]. Similarly, the findings in Hernández-Vásquez et al. [16] and Garg et al. [20] suggest that having health insurance cover did not provide adequate financial protection during the COVID-19 pandemic. Furthermore, shifts in healthcare utilization to the private sector were noted in some countries, possibly causing financial hardship [15,20]. The pandemic can thus be considered a stress test for financial protection of universal health coverage.

Prior work on the association of health insurance type with healthcare utilization and OOP tends to focus on health systems in which universal population coverage has not yet been achieved [21–24], and not on contexts with universal population coverage and fragmented pooling. Fragmented pooling limits redistributive capacity and can result in inequitable access to health services as well as differences in OOP [25], especially when health systems are under stress, which to our knowledge has not yet been examined in the context of the COVID-19 pandemic. To fill this gap in research, data from the 2019 and 2021 waves of the Health and Welfare Survey (HWS) are used in this study to examine the determinants of healthcare utilization and OOP in Thailand where universal population coverage was achieved in 2002 while maintaining fragmentation in pooling. In terms of methodologies, compared to the probability of utilizing healthcare services, OOP are more difficult to model because of their unique statistical features. Several alternative models have been suggested in the literature to cope with these depending on the specific characteristics of the data [26,27]. This study contributes to the literature by (i) focusing on the type of public health insurance given prevailing fragmentation, (ii) using data collected during pre- and peri-pandemic time periods, (iii) examining the interplay between different types of public health insurance and the period before and during the COVID-19 pandemic, and (iv) comparing two alternative models for OOP, the two-part model and the Tobit model.

## Methods

### HWS overview and ethical approval

The HWS is a nationally representative survey conducted biannually by the National Statistical Office of Thailand using a stratified two-stage sampling design. Seventy-seven provinces, divided into municipal and non-municipal areas except Bangkok, are used as stratification variables. Primary sampling units (i.e., enumeration areas within the strata) are sampled using the proportional-to-size method, while systematic random sampling is used to select secondary sampling units (i.e., households in each selected enumeration area) [28,29]. The questionnaire designed by the National Statistical Office of Thailand elicits information on household and respondent characteristics, health status, recent illnesses and accidents, access to and utilization of health services.

Permission to use the proprietary HWS data was granted by the National Statistical Office of Thailand under contract number 30/2566. Approval to conduct this study, on the other hand, was granted by the Research Ethics Review Committee for Research Involving Human Subjects: The Second Allied Academic Group in Social Sciences, Humanities and Fine and Applied Arts at Chulalongkorn University (Certificate of Research Approval No. 240/66). The HWS data are de-identified, third-party data and informed consent for secondary data analysis is not required.

### Participants

A repeated cross-sectional research design was used and participants were drawn from the 2019 and 2021 waves of the HWS. The 2021 data were collected in March, that is between the second and third COVID-19 waves, while the 2019 data were collected before the pandemic. Although Thailand was the first country outside of China to report a COVID-19 case, stringent measures taken early on helped to contain the first wave. A second wave occurred between December 2020 and February 2021, with a peak of 959 new daily cases at the end of January 2021 [30]. In April 2021, a third COVID-19 wave began to unfold. The cumulative number of new cases was still relatively low in March 2021 when the HWS was conducted. The 2019 and 2021 HWS datasets contain information from 27,960 households with 63,594 (2019) and 63,780 (2021) individuals. In this study only data from Thai individuals (i) aged 18 years and above, (ii) covered by one of the main three public health insurance schemes, and (iii) with self-reported illness in the past month before the data collection were used. Furthermore, data from respondents with a chronic disease who reported having had an illness in the past month were only used for the OOP analyses but not for the healthcare utilization analyses since they all visited a healthcare provider. After data cleaning, the sample used to analyze outpatient care utilization comprises 10,220 individuals, while data from 12,014 individuals are used for the analysis of OOP. The number of observations differs between the utilization and OOP analyses because they are conducted separately. The utilization analyses include individuals who experienced illness in the past month, excluding those with chronic conditions, to examine their decision to use outpatient care and their choice of healthcare provider. In contrast, the OOP analyses include all individuals who had an outpatient visit, including those with chronic illnesses, to capture their out-of-pocket expenses from their most recent visit.

### Data analysis

#### Healthcare utilization.

The probability of using outpatient services is modelled using a logit. The binary dependent variable takes the value of one to indicate the use of outpatient services in the past month and zero otherwise. Healthcare utilization is subsequently further broken down into three categories analogous to Paek et al. [31], that is (i) no use of formal outpatient services (including self-care and care received from traditional/local healers), (ii) using health services at a non-designated facility, and (iii) using health services at a designated facility. A multinomial logit model is employed to analyze this choice. Designated facilities are facilities at which insureds can seek healthcare services under their public health insurance scheme. While the HWS dataset does not provide information about non-designated and designated facilities per se, information about the use of public health insurance for the last treatment is available. Insureds who used their public health insurance for the last treatment are assumed to have visited a designated facility. Conversely, CSMBS, SSS, or UCS beneficiaries who opted not to use their public health insurance for their last visit are assumed to have visited a non-designated provider. SSS and UCS benefits can only be accessed at public and private facilities listed in the provider registry of their health insurance scheme (i.e., designated facilities). Similarly, CSMBS benefits are restricted to services received from public providers (Table A1 in S1 Appendix). Distinguishing between designated and non-designated facilities is important as respondents can choose to forgo benefits under their public health insurance scheme by visiting a non-designated provider.

#### Out-of-pocket health expenditure.

For the analysis of OOP, two-part models are estimated given the skewness of the dependent variable and its large mass at zero [26]. The dependent variable is OOP in Thai Baht (THB) for the last outpatient treatment in the past month (decomposed into medical and transportation expenditures). Analogous to the literature [32,33], both medical spending (i.e., medical spending not covered by a health insurance scheme) and nonmedical spending (i.e., spending on travel to access healthcare) are considered. Medical and transportation expenditures are top-coded at 9,998 THB in the HWS and the latter was further top-coded at THB 5,000 to remove extreme values. The exchange rate is approximately 34 THB per United States dollar. Of those individuals who used outpatient services, 5,749 incurred positive medical expenditures and 9,814 positive transportation expenditures. The probability of incurring positive OOP is modelled by a logit (first part), while a generalised linear model (GLM) with a log link function is used for payments made by the subset of individuals with positive OOP (second part). Standard tests were used to choose link function and distribution family for the GLM. OOP for the last inpatient episode over a 12-month recall period are subsequently also examined, mainly to corroborate key results. The sample size is substantially smaller as a different sample is used (n=3,613). OOP for the last inpatient episode are top-coded at THB 99,998 (medical) and THB 9,999 (transportation) in the HWS survey. In contrast to outpatient services, the utilization of inpatient services largely depends on clinicians and is thus not examined.

The two-part model was advocated in the 1980s [27]. Manning et al. [34], for example, used the first equation of the model for the analysis of healthcare utilization and the second equation for the analysis of the conditional utilization frequency. Subsequent adaptations of the two-part model to healthcare expenditure data either (i) modelled the utilization choice in the first step and the conditional expenditure magnitude in the second step [e.g., 35], or (ii) first modelled the probability of having any health expenditures and then used a conditional regression model for positive amounts spent [e.g., 26]. This study follows the second approach. Healthcare utilization and out-of-pocket payments are modelled separately in this study given that the fraction of the sample that did not incur any OOP comprises users and non-users of outpatient services.

In addition, an alternative modelling approach is employed, namely a Type 1 Tobit model as categorised by Amemiya [36] to estimate the out-of-pocket medical and travel expenditure. This Tobit model treats OOP as left-censored data with the censoring point at zero. The main assumptions underlying the model are that the same latent index determines both the decisions to spend as well as the amount of OOP, and that the error term is normally distributed. As extensively discussed in Jones [27], some researchers favour the Tobit model while others strongly advocate the use of the two-part model over the Tobit model. Arguments put forward suggest that preference should be given to the two-part model in this study because the OOP data have a large fraction of zero observations. The decision to spend and the decision on how much to spend are likely to be sequential rather than a joint decision. Moreover, there is also no obvious variable in the data to be used for the exclusion restriction when considering the Tobit model.

#### Independent variables.

Public health insurance status is the main variable of interest. Binary variables are created for public health insurance status, taking the value of 1 if the individual is covered by the CSMBS (variable CSMBS) or the SSS (variable SSS) and 0 otherwise. To see if the association between health insurance status and outcome variables differs by survey year, interaction terms between health insurance status and a year dummy variable are subsequently added to the models.

The explanatory variables are based on the Andersen-Newman framework of health services utilization [37]. These include gender, age and marital status (pre-disposing factors), education, income, location, public and private health insurance coverage (enabling factors), and having a chronic disease (need factor). Since some individuals did not use their public health insurance for the last treatment, an additional binary variable for those who “opted out” on this occasion is used for the analysis of OOP.

Minor illness was recorded as the main reason for opting out (62.14%). About one third of those who opted out had concerns about covered services such as, for example, long waiting times (18.77%). Furthermore, having an individual private health insurance may indicate partial opting out and reflect dissatisfaction with covered services [38]. The reasons for opting-out, however, do not differ much by health insurance status.

Variables, their definitions and summary statistics are shown in Table 1. Sample weights provided in the dataset are applied in all regressions. All statistical analyses are performed with Stata version 14.2.

**Table 1:** Variable definitions and summary statistics.

Variables	Definition	Mean/pct
utilization^a^	utilization of outpatient services in the past month: 1 = used outpatient services, 0 = otherwise	0.466
utilization choice^a^	three outcome categories that take the value of 1, 2 and 3	
informal care	self-care, and care received from traditional/local healers (base outcome)	0.534
formal healthcare at non-designated facility	received formal healthcare but did not use the benefits provided by the public health insurance	0.111
formal healthcare at designated facility	received formal healthcare and used the benefits provided by the public health insurance	0.355
out-of-pocket health expenditures	out-of-pocket health expenditure in Thai Baht related to the last outpatient treatment in the past month	
medical	medical expenditures	146.02
transportation	transportation expenditures	118.82
female	gender: 1 = female, 0 = male	0.604
age	age in years at the time the survey was conducted	58.096
married	marital status: 1 = married, 0 = other marital status (e.g., single, widowed, divorced)	0.628
education	no formal education, primary education, lower and upper secondary education, higher education	
upper secondary education	1 = upper secondary education, 0 = otherwise	0.098
higher education	1 = higher education, 0 = otherwise	0.101
income	household income divided by the square root of household size (log-transformed)^b^	8.863
municipal area	area: 1 = municipal area, 0 = non-municipal area	0.525
region	central (including Bangkok), north, northeast, south	
north	1 = north, 0 = otherwise	0.250
northeast	1 = northeast, 0 = otherwise	0.275
south	1 = south, 0 = otherwise	0.155
public health insurance scheme	CSMBS, SSS, UCS	
CSMBS	1 = covered by CSMBS, 0 = otherwise	0.098
SSS	1 = covered by SSS, 0 = otherwise	0.085
dual cover	individual private health insurance: 1 = yes, 0 = no	0.049
opt out	1 = did not use the benefits provided by the public health insurance for last treatment, 0 = otherwise	0.444
chronic illness	presence of chronic/congenital illness: 1 = yes, 0 = no	0.558
year 2021	year of data collection: 1 = 2021, 0 = 2019	0.469
observations		12,014

*Note.* n = 12,014 (except for utilization and utilization choice). Reported are means for continuous variables and percentages (pct) for categorical variables, based on unweighted data. Mean variance inflation factor: 1.27.

^a^n = 10,220.

^b^Equivalence scale based on the 2013 OECD framework for statistics on the distribution of household income, consumption and wealth.

## Results

Estimation results from the healthcare utilization regression models are presented in Table 2. Type of health insurance does either not have an effect on healthcare utilization (CSMBS) or only a weak effect (SSS). Similarly, the coefficient of the year dummy variable is insignificant suggesting that the COVID-19 pandemic did not impact the utilization of outpatient services in February/March 2021. Female, older, married and better-educated individuals as well as individuals with a chronic illness have higher odds of using outpatient services in case of illness. The likelihood of healthcare utilization, on the other hand, is found to be lower for individuals with higher income as well as those living in municipal areas. In addition, there are some regional differences. The results for the interaction terms between health insurance status and the dummy variable for the year 2021 (Table 2, column 2) do not indicate a stronger effect of health insurance type on healthcare utilization in 2021. The likelihood ratio test result shows though that the interaction terms are statistically insignificant overall. The results obtained from estimating the multinomial model (Table 2, columns 3 and 4), on the other hand, suggest that health insurance status affects provider choice. Individuals covered by the CSMBS or the SSS are more likely to seek outpatient services from designated providers and less likely to turn to non-designated providers relative to the base outcome. The statistically significant positive effect of health insurance status on the use of designated providers, however, largely disappears when interaction terms between health insurance status and the dummy variable for the year 2021 are added (Table 2, column 6). Notwithstanding, the interaction terms are not simultaneously statistically significant based on the likelihood ratio test.

**Table 2:** Healthcare utilization (outpatient services).

	(1)	(2)	(3)	(4)	(5)	(6)
	binomial	binomial	multinomial		multinomial	
	utilization	utilization	non-designated facility	designated facility	non- designated facility	designated facility
female	0.183***	0.182***	0.371***	0.124*	0.373***	0.123*
	(0.06)	(0.06)	(0.10)	(0.06)	(0.10)	(0.06)
age	0.004*	0.004*	−0.001	0.005**	−0.001	0.005**
	(0.00)	(0.00)	(0.00)	(0.00)	(0.00)	(0.00)
married	0.118*	0.113*	0.381***	0.046	0.381***	0.041
	(0.06)	(0.06)	(0.10)	(0.07)	(0.10)	(0.07)
upper secondary education	0.214**	0.215**	0.310**	0.180	0.308**	0.181
	(0.10)	(0.10)	(0.16)	(0.11)	(0.16)	(0.11)
higher education	0.384***	0.389***	0.686***	0.286**	0.686***	0.291**
	(0.12)	(0.12)	(0.16)	(0.13)	(0.16)	(0.13)
income	−0.122***	−0.121***	0.079	−0.179***	0.079	−0.179***
	(0.04)	(0.04)	(0.08)	(0.05)	(0.08)	(0.05)
municipal area	−0.213***	−0.215***	−0.332***	−0.180***	−0.331***	−0.183***
	(0.06)	(0.06)	(0.10)	(0.06)	(0.10)	(0.06)
north	0.104	0.109	0.398***	0.016	0.395***	0.022
	(0.08)	(0.08)	(0.12)	(0.08)	(0.12)	(0.08)
northeast	0.595***	0.597***	0.600***	0.597***	0.598***	0.598***
	(0.08)	(0.08)	(0.13)	(0.08)	(0.13)	(0.08)
south	0.350***	0.354***	0.601***	0.267***	0.600***	0.271***
	(0.09)	(0.09)	(0.14)	(0.09)	(0.14)	(0.09)
CSMBS	0.029	0.022	−0.806***	0.248**	−0.646**	0.212
	(0.12)	(0.15)	(0.20)	(0.12)	(0.25)	(0.16)
SSS	0.174*	0.038	−0.363**	0.337***	−0.363*	0.164
	(0.11)	(0.13)	(0.18)	(0.11)	(0.21)	(0.15)
dual cover	0.078	0.076	0.855***	−0.413**	0.850***	−0.414**
	(0.14)	(0.14)	(0.18)	(0.18)	(0.18)	(0.18)
chronic illness	0.698***	0.700***	0.523***	0.748***	0.523***	0.749***
	(0.06)	(0.06)	(0.11)	(0.07)	(0.11)	(0.07)
year 2021	−0.057	−0.105*	−0.177*	−0.021	−0.162	−0.085
	(0.06)	(0.06)	(0.10)	(0.06)	(0.10)	(0.07)
CSMBS*year 2021		0.014			−0.449	0.075
		(0.20)			(0.34)	(0.22)
SSS*year 2021		0.308			−0.013	0.379*
		(0.19)			(0.34)	(0.20)
constant	0.11	0.13	−3.163***	0.346	−3.166***	0.375
	(0.43)	(0.43)	(0.74)	(0.46)	(0.74)	(0.46)
n	10,220	10,220	10,220		10,220	
HL (p-value)	>0.10	>0.10	>0.10		>0.10	
LR (p-value)		>0.10			>0.10	
base outcome			no or informal treatment		no or informal treatment	

*Note*. Standard errors in parentheses. HL: Hosmer–Lemeshow test. LR: likelihood ratio test.

* p < 0.10, ** p < 0.05, *** p < 0.01

Turning to control variables, the results in Table 2 (columns 3 and 4) inter alia show that having higher education, an individual private health insurance, and a chronic illness increases the probability of visiting a non-designated provider relative to the base outcome, while living in municipal areas decreases it. Chronic illness is also positively associated with using a designated facility. In addition, there is weak evidence that the odds of visiting a non-designated facility were lower in 2021.

Table 3 presents the results from estimating the two-part model. Having CSMBS or SSS coverage decreases the probability of incurring positive out-of-pocket medical spending for outpatient services compared to UCS beneficiaries. Conditional on having positive expenditures, CSMBS beneficiaries have lower medical but higher transportation expenditures. SSS beneficiaries, on the other hand, spend less on transportation, conditional on positive expenditure. The combined marginal effects indicate that the magnitude of these effects is relatively small though. Nonetheless, it is important to point out that the mean household income of UCS beneficiaries, a reference health insurance category, is substantially lower than that of CSMBS and SSS beneficiaries. In other words, the UCS group faces slightly higher medical expenses along with a lower median income, resulting in a greater share of their income being spent on healthcare costs in comparison to the CSMBS and SSS groups. The statistically significant effect of health insurance status on amounts spent for travel disappears, however, when interaction terms between health insurance status and the year dummy variable are added (Table 4). The likelihood ratio test statistics show that the interaction terms are jointly significant. Table 4 thus contains the appropriate specification of the OOP model. The combined marginal effects indicate that CSMBS beneficiaries paid THB 79.4 less in medical expenses. The results in Table 4 further show that CSMBS beneficiaries spent more on travel in 2021, conditional on spending. Moreover, the coefficients on the year dummy variable suggest that the probability of positive medical spending and, conditional on any spending, amounts spent on travel were lower in 2021. When transportation expenditure is used as dependent variable, however, the modified Hosmer-Lemeshow statistic is statistically significant at the 5% level suggesting that the model is not a good fit.

**Table 3:** Two-part model for out-of-pocket health expenditures (outpatient services).

	(1)	(2)	(3)	(4)	(5)	(6)
	first part		second part		combined marginal effects	
	medical	travel	medical	travel	medical	travel
female	−0.009	−0.005	0.010	0.023	1.146	2.708
	(0.08)	(0.07)	(0.08)	(0.04)	(12.19)	(4.66)
age	−0.018***	−0.012***	0.011***	0.003**	1.120**	0.134
	(0.00)	(0.00)	(0.00)	(0.00)	(0.45)	(0.15)
married	0.166*	0.306***	0.075	0.107***	16.665	17.361***
	(0.09)	(0.08)	(0.09)	(0.04)	(12.89)	(4.80)
secondary education	−0.177	0.136	0.189	0.124*	24.497	17.822**
	(0.15)	(0.13)	(0.12)	(0.06)	(20.60)	(8.93)
higher education	−0.132	0.328**	0.474***	0.225***	80.538***	34.965***
	(0.16)	(0.15)	(0.13)	(0.06)	(28.12)	(8.99)
income	0.222***	0.122**	0.222***	0.070***	40.958***	10.261***
	(0.06)	(0.05)	(0.05)	(0.03)	(9.07)	(3.19)
municipal area	0.154*	−0.104	0.070	−0.162***	15.519	−20.942***
	(0.09)	(0.07)	(0.09)	(0.04)	(13.33)	(4.68)
north	−0.218*	0.396***	−0.137	−0.100*	−26.552*	−6.006
	(0.12)	(0.09)	(0.11)	(0.05)	(15.69)	(6.15)
northeast	−0.174	0.054	0.138	0.210***	15.912	27.011***
	(0.12)	(0.09)	(0.12)	(0.05)	(19.25)	(7.35)
south	0.248**	1.397***	0.164	0.030	35.500*	20.376***
	(0.12)	(0.13)	(0.11)	(0.05)	(19.87)	(6.25)
CSMBS	−0.937***	−0.016	−0.304**	0.183***	−65.614***	22.989***
	(0.14)	(0.14)	(0.12)	(0.06)	(14.38)	(8.82)
SSS	−0.808***	−0.200	−0.034	−0.097*	−29.896*	−14.129**
	(0.14)	(0.13)	(0.13)	(0.06)	(17.31)	(6.41)
dual cover	0.032	−0.094	0.367***	0.351***	66.838**	46.911***
	(0.20)	(0.17)	(0.14)	(0.07)	(30.06)	(12.68)
opt out	3.997***	−1.859***	0.349***	−0.396***	299.372***	−76.464***
	(0.09)	(0.08)	(0.13)	(0.04)	(22.82)	(4.48)
chronic illness	0.004	0.391***	0.877***	0.445***	137.746***	55.657***
	(0.09)	(0.08)	(0.09)	(0.04)	(19.04)	(5.00)
year 2021	−0.226***	−0.086	0.013	−0.077**	−5.431	−10.508**
	(0.08)	(0.07)	(0.08)	(0.04)	(12.16)	(4.51)
constant	−2.508***	1.578***	2.173***	3.948***		
	(0.60)	(0.50)	(0.55)	(0.25)		
n	12,014					
MLH (p−value)	>0.10	<0.01				

*Note.* Standard errors in parentheses. MHL: modified Hosmer–Lemeshow test. * p < 0.10, ** p < 0.05, *** p < 0.01.

**Table 4:** Two−part model for out−of−pocket health expenditures (outpatient services) with interaction terms.

	(1)	(2)	(3)	(4)	(5)	(6)
	first part		second part		combined marginal effects	
	medical	travel	medical	travel	medical	travel
female	−0.011	−0.003	0.006	0.024	0.567	2.756
	(0.08)	(0.07)	(0.08)	(0.04)	(12.18)	(4.66)
age	−0.018***	−0.012***	0.011***	0.003**	1.113**	0.130
	(0.00)	(0.00)	(0.00)	(0.00)	(0.45)	(0.15)
married	0.163*	0.308***	0.075	0.108***	16.531	17.510***
	(0.09)	(0.08)	(0.09)	(0.04)	(12.91)	(4.80)
secondary education	−0.174	0.134	0.190*	0.126*	24.761	18.069**
	(0.15)	(0.13)	(0.11)	(0.06)	(20.15)	(8.91)
higher education	−0.128	0.328**	0.471***	0.219***	80.086***	34.166***
	(0.15)	(0.16)	(0.13)	(0.06)	(28.26)	(8.83)
income	0.222***	0.121**	0.221***	0.069***	40.886***	10.162***
	(0.06)	(0.05)	(0.05)	(0.03)	(9.06)	(3.20)
municipal area	0.151*	−0.102	0.065	−0.159***	14.669	−20.597***
	(0.09)	(0.07)	(0.08)	(0.04)	(13.19)	(4.69)
north	−0.214*	0.394***	−0.136	−0.103**	−26.267*	−6.398
	(0.12)	(0.09)	(0.11)	(0.05)	(15.74)	(6.15)
northeast	−0.172	0.053	0.138	0.209***	15.850	26.920***
	(0.12)	(0.09)	(0.12)	(0.05)	(19.26)	(7.33)
south	0.251**	1.395***	0.164	0.029	35.576*	20.271***
	(0.12)	(0.13)	(0.11)	(0.05)	(19.85)	(6.22)
CSMBS	−1.002***	0.118	−0.421***	0.065	−79.425***	9.831
	(0.17)	(0.19)	(0.14)	(0.08)	(15.61)	(10.44)
SSS	−0.935***	−0.147	−0.017	−0.053	−31.635	−8.482
	(0.16)	(0.17)	(0.17)	(0.07)	(21.42)	(8.69)
dual cover	0.033	−0.098	0.373***	0.346***	68.150**	46.037***
	(0.20)	(0.17)	(0.14)	(0.07)	(30.13)	(12.30)
opt out	4.001***	−1.861***	0.351***	−0.398***	300.177***	−76.754***
	(0.09)	(0.08)	(0.13)	(0.04)	(22.87)	(4.49)
chronic illness	0.005	0.389***	0.878***	0.446***	137.972***	55.680***
	(0.09)	(0.08)	(0.09)	(0.04)	(19.03)	(4.98)
year 2021	−0.273***	−0.051	0.005	−0.073*	−8.146	−9.484*
	(0.09)	(0.08)	(0.09)	(0.04)	(13.63)	(5.18)
CSMBS*year 2021	0.138	−0.276	0.264	0.233**	51.279	25.048
	(0.25)	(0.25)	(0.20)	(0.11)	(40.93)	(16.47)
SSS*year 2021	0.282	−0.118	−0.037	−0.099	3.437	−12.954
	(0.22)	(0.24)	(0.24)	(0.09)	(37.70)	(10.67)
constant	−2.496***	1.568***	2.186***	3.955***		
	(0.60)	(0.50)	(0.55)	(0.25)		
n	12,014					
MLH (p−value)	>0.05	<0.05				
LR (p−value)	<0.01	<0.01				

*Note.* Standard errors in parentheses. LR: likelihood ratio test. MHL: modified Hosmer–Lemeshow test. * p < 0.10, ** p < 0.05, *** p < 0.01.

Turning to other control variables for the two−part model estimation in Table 4, older persons are less likely to incur out−of−pocket medical and transportation expenditures for outpatient services. Marital status and income, on the other hand, increase the likelihood of having positive medical and transportation expenditures, while higher education and having a chronic illness is positively associated with the probability of spending for transportation. In addition, there are regional differences in the probability of incurring positive OOP. Age, more education, income, having a private health insurance and chronic illness are positively related to out−of−pocket spending for outpatient services, conditional on spending. Focusing on the need factor, the combined marginal effects show that those with chronic illness have THB 138.0 more in medical spending and THB 55.7 more in spending on travel than those without. Married persons spend more on transportation, whereas those living in municipal areas spend less, conditional on spending. Opting out of public health insurance increases probability and magnitude of medical spending, but is negatively associated with the odds of paying for travel as well as the magnitude thereof.

The Tobit results for CSMBS and SSS appear to be consistent with those from the two−part model (Tables A2 and A3 in S1 Appendix). The signs of the estimated coefficients are exactly the same although the magnitudes of the marginal effects are slightly larger for the Tobit model. For example, compared to UCS beneficiaries, CSMBS and SSS beneficiaries on average had lower out−of−pocket medical expenditure by THB 103.3 and THB 59.3 under the Tobit estimation versus THB 65.6 and THB 29.9 under the two−part model estimation, respectively. When adding interaction terms to the model (Table A3 in S1 Appendix), the same findings generally hold. However, the impact of the insurance variables on travel expenditure appears to be less significant. Additionally, the coefficients associated with the interaction terms between health insurance variables and the dummy variable for the year 2021 are all found to be statistically insignificant, which is essentially the same finding as in case of the two−part model. The alternative Tobit model also finds the impact of most control variables to be comparable to the two−part model. Given that the main results from the two−part model and the Tobit model are closely similar, it can be implied that regardless of whether a model incorporates the correlation between the decision to spend and the amount to spend as in the Tobit model or does not take it into account as in the two−part model there appears to be no major discrepancy in the estimation results when applied to the HWS data. Essentially, this comparison of the results helps to reassure that our preferred two−part model that allows for greater flexibility can be accurately used in this study even in the midst of the debates over alternative models for healthcare expenditures [27,39].

To further corroborate the results related to health insurance status and the dummy variable for 2021, the two−part model is estimated for out−of−pocket spending on inpatient services (Table A4 in S1 Appendix). The association between health insurance status and the likelihood of having positive expenditures for inpatient services is statistically insignificant. Conditional on positive expenditures, CSMBS and SSS beneficiaries spend more on travel related to inpatient stays. Moreover, the probability of incurring OOP for inpatient services was lower in 2021 compared to 2019.

## Discussion

The majority of the Thai population is covered by one of the three main health insurance schemes, that is the CSMBS, the SSS and the UCS. Through the implementation of the UCS, near universal population coverage was achieved within a short period of time. The financing system is fragmented though and pool members’ health risks differ across pools. Fragmentation can result in inequitable access to health services as well as differences in out−of−pocket health expenditures [25], especially during times of health system stress such as during the COVID−19 pandemic.

The present study shows that the type of health insurance is not (or only weakly) associated with outpatient healthcare utilization, suggesting that fragmentation does not lead to disparities in healthcare access. This result is contrary to studies conducted in China [23,40] and South Korea [41]. At the same time, it underlines the impressive achievement of Thailand’s universal health coverage more than two decades after the introduction of the UCS, especially in the face of the COVID−19 pandemic. Although the annual per−capita expenditure is much higher in case of the CSMBS than the UCS [42], for example, no statistically significant difference between CSMBS and UCS beneficiaries in outpatient service utilization was found in the present study. Yet, the type of health insurance is found to affect the choice of provider (non−designated versus designated) in this study, which could be due to the primary care level gatekeeping system in the UCS. Provider networks, often in the form of district health systems consisting of health centres and a district hospital, serve as gatekeepers for the UCS. Patients are required to visit the provider network that they are registered with in order to access higher levels of care [8]. In January 2025, however, the “30−Baht Treatment Anywhere” policy which started in 2024 was implemented nationwide, permitting UCS beneficiaries to access healthcare providers registered with the UCS other than their designated providers [43]. CSMBS beneficiaries, who enjoy free public provider choice, and SSS beneficiaries, who are required to go to the hospital they are registered with, are less (more) likely to visit a non−designated (designated) provider relative to informal care than UCS beneficiaries. In other words, UCS beneficiaries are more likely to opt−out from using their public health insurance in comparison to their CSMBS and SSS counterparts when undergoing outpatient treatment. This result may reflect concerns about covered services under the UCS scheme [31]. The reasons provided in the HWS dataset for not using a public health insurance for the last treatment received, however, do not permit such conclusion.

Focusing on OOP for outpatient services, CSMBS beneficiaries were estimated to have paid a bit less in medical expenditure for the last treatment than UCS beneficiaries. Similarly, the type of health insurance only had a small effect on out−of−pocket spending on travel related to inpatient services but not medical spending. These results further accentuate the continued success of Thailand’s universal health coverage in terms of limited or no OOP, especially peri−COVID−19, and confirm the findings of the descriptive statistical analysis by Damrongplasit et al. [44] as well as Tangcharoensathien et al. [14]. Studies that focus on health insurance type, however, generally examine its effect on out−of−pocket spending relative to being uninsured and their results are mixed [21−–24]. Important correlates such as age, education and income also confirm the evidence in the existing literature on the determinants of OOP [12,13,35,45].

### Strengths and limitations

This study focuses on Thailand’s universal health coverage system, which has achieved impressive results while maintaining fragmentation in health financing. It uses nationally representative survey data, and to our knowledge is the first study that investigates the effect of public health insurance type on outpatient healthcare utilization and out−of−pocket expenditure, pre− and peri−COVID−19. The general drawback of secondary data, however, is the limited choice of variables. Moreover, it is important to bear in mind that the cross−sectional nature of the HWS data as well as possible selection bias (e.g., unhealthy respondents may be more likely to become civil servants given the more generous nature of the CSMBS) do not allow causal inference. The results must, therefore, be interpreted cautiously. Second, with respect to the notion of limited or no OOP, it needs to be emphasised that this study only considers the last visit to a healthcare provider and does not account for multiple visits which will burden vulnerable population groups more than other groups [46]. Moreover, despite the outstanding achievements of Thailand’s universal health coverage, manifold challenges have remained, including, but not limited to (i) securing more fiscal space, (ii) developing alternative financing strategies, (iii) reducing the inequity caused by the CSMBS’ higher per−capita expenditure, and (iv) accommodating changing health needs [47]. Besides, other challenges associated with fragmentation such as inefficiencies arising from a duplication of tasks across multiple pools should be considered as well.

### Policy implications

One potential reason explaining the small effect of public health insurance status on outpatient healthcare utilization and OOP, even during a health crisis like COVID−19, could be the progressive development of Thailand’s universal health coverage system since its inception and efforts to harmonize the main public health insurance schemes. This explanation lends support to the policy recommendation in Tangcharoensathien et al. [48] that low and middle−income countries should seek early harmonization across schemes to address fragmentation in health financing.

While out−of−pocket spending has remained low in Thailand, the results nonetheless point to a burden that people with chronic illness and higher need more generally are facing. This is a cause for concern given that more care is sought by those with chronic diseases and that the number of persons with chronic diseases will increase in the future. Having a chronic illness has been identified in the literature as a strong correlate of OOP [49,50]. Policymakers may consider encouraging the take−up of supplementary private health insurance to mitigate the burden on high−need individuals. However, those who opt out and those with additional private health insurance are found to spend more out−of−pocket for services received, possibly because they choose to visit private facilities or use services that are more expensive, which is in line with the literature [51,52]. Alternatively, policymakers could continue expanding UCS coverage to include high−cost services, as they have done in the past, to further enhance financial protection for individuals with chronic conditions and with greater healthcare needs.

In addition, there are regional differences as well as differences between municipal and non−municipal areas, especially in terms of healthcare utilization and spending on travel, that should be explored further given that the geographical distribution of some healthcare resources has remained unequal [47].

## Conclusion

Using repeated cross−sectional national survey data, collected before and during the coronavirus pandemic, this study empirically examines the determinants of outpatient healthcare utilization and OOP in Thailand, focusing on type of public health insurance given existing fragmentation. The findings show that in terms of outpatient healthcare utilization as well as financial protection, Thailand’s universal health coverage system has continued to live up to its promise, also during the early stages of the COVID−19 pandemic. Notwithstanding, this study highlights that universal health coverage is an ongoing effort that requires careful monitoring, inter alia to mitigate undesirable consequences of fragmentation and to ensure that high−need and other vulnerable persons are not left behind. Thailand’s experience pre− and peri−COVID−19 further strengthens the case for universal health coverage and its centrality for achieving the Sustainable Development Goals.

## Supporting information

S1 Appendix Tables A1−A4.
(DOCX)
