# Supplementary material for: Health insurance type, healthcare utilization and out-of-pocket expenditure in the face of COVID-19: Evidence from Thai national survey data
Source: PLoS One. 2025 Apr 8;20(4):e0321468. doi: 10.1371/journal.pone.0321468 (PMC11977970; doi:10.1371/journal.pone.0321468)
Supplement: S1 Appendix — Tables A1−A4. (DOCX) [file pone.0321468.s001.docx]

Health insurance type, healthcare utilization and out-of-pocket expenditure in the face of COVID-19: Evidence from Thai national survey data

S1 Appendix

Table A1 Overview of Thailand’s main health insurance schemes

|  | CSMBS (since 1980) | SSS (since 1990) | UCS (since 2002) |
| --- | --- | --- | --- |
| Revenue source | general taxes (annual budget allocation) | tripartite contributions (by employees, employers, and the government) | general taxes (annual budget allocation) |
| Administration | Comptroller General’s Department, Ministry of Finance | Social Security Office | National Health Security Office |
| Population coverage (2017) 1/ | 7.89%  (civil servants, their dependants as well as retired civil servants) | 18.64%  (formal sector employees aged 15 to 60 years) | 71.13% |
| Benefit package 2/ | comprehensive (use of medicines not included in the National List of Essential Medicines possible) | comprehensive | comprehensive (health promotion and disease prevention services for the Thai population) |
| Providers | free choice of public provider | registered (designated) public or private providers | registered (designated) public or private providers, gatekeeping |
| Provider payment | - capitation (OP) - DRG (IP) | - capitation (OP, IP) - DRG (IP if high cost, i.e. if adjusted risk weight > 2) | - capitation (OP, PP) - DRG with global budget (IP) - fee schedule (specific services/conditions) |
| Emergency medical services 3/ | Universal Coverage for Emergency Patients since 2017: free emergency treatment in the nearest public or private hospital for up to 72 hours | | |

*Note*. There are other specific health insurance schemes for defined population groups such as, for example, local administration officers (1.09%). The remaining population with a right to universal health coverage comprises (i) persons effectively stateless (0.81%), (ii) persons with unknown citizen status (0.14%), and (iii) persons qualified but not registered with the UCS (0.28%). The information is from Viriyathorn et al. [1], and from 1/ National Health Security Office [2], 2/ Patcharanarumol et al. [3], as well as 3/ Satchanawakul [4]. CSMBS: Civil Servant Medical Benefit Scheme, DRG: diagnosis-related group, IP: inpatient services, OP: outpatient services, PP: health promotion and disease prevention services, SSS: Social Security Scheme, UCS: Universal Coverage Scheme.

Table A2 Tobit model for censored out-of-pocket health expenditures (outpatient services)

|  | (1) | (2) | (3) | (4) |
| --- | --- | --- | --- | --- |
|  | coefficients | | marginal effects | |
|  | medical | travel | medical | travel |
| female | -0.851 | -0.704 | -0.297 | -0.421 |
|  | (28.06) | (7.11) | (9.80) | (4.26) |
| age | -0.886 | -0.456* | -0.31 | -0.273* |
|  | (1.14) | (0.27) | (0.40) | (0.16) |
| married | 32.887 | 24.098*** | 11.439 | 14.328*** |
|  | (28.82) | (7.31) | (9.98) | (4.26) |
| secondary education | 9.69 | 13.949 | 3.396 | 8.441 |
|  | (39.23) | (9.53) | (13.80) | (5.85) |
| higher education | 123.177* | 58.703*** | 45.011* | 36.821*** |
|  | (66.61) | (14.34) | (25.41) | (9.09) |
| income | 97.159*** | 19.575*** | 33.933*** | 11.713*** |
|  | (21.43) | (5.42) | (7.50) | (3.14) |
| municipal area | 46.983* | -23.849*** | 16.464* | -14.208*** |
|  | (27.96) | (8.01) | (9.83) | (4.64) |
| north | -71.202** | 11.581 | -24.349** | 6.981 |
|  | (35.50) | (8.65) | (11.89) | (5.21) |
| northeast | -5.6 | 31.115*** | -1.953 | 18.891*** |
|  | (36.15) | (9.06) | (12.59) | (5.50) |
| south | 47.591 | 62.923*** | 16.909 | 39.536*** |
|  | (33.56) | (17.12) | (12.11) | (10.69) |
| CSMBS | -343.963*** | 53.052*** | -103.266*** | 33.307*** |
|  | (60.70) | (19.59) | (15.92) | (12.52) |
| SSS | -180.978*** | -23.187** | -59.297*** | -13.621** |
|  | (50.27) | (10.23) | (15.45) | (5.85) |
| dual cover | 87.134 | 71.791** | 31.604 | 45.908** |
|  | (64.60) | (33.49) | (24.33) | (22.11) |
| opt out | 1208.838*** | -136.133*** | 424.485*** | -80.685*** |
|  | (87.51) | (10.58) | (28.87) | (5.06) |
| chronic illness | 233.600*** | 77.328*** | 82.340*** | 46.282*** |
|  | (38.98) | (8.69) | (13.72) | (4.85) |
| year 2021 | -58.591** | -9.12 | -20.410** | -5.453 |
|  | (28.93) | (6.87) | (10.03) | (4.13) |
| constant | -1872.468*** | -86.276 |  |  |
|  | (250.08) | (60.02) |  |  |
| sigma | 842.711*** | 281.202*** |  |  |
|  | (54.42) | (22.86) |  |  |
| n | 12,014 | 12,014 | 12,014 | 12,014 |
| *Note.* * p<0.10, ** p<0.05, *** p<0.01 | |  |  |  |

Table A3 Tobit model for censored out-of-pocket health expenditures (outpatient services) with interaction terms

|  | (1) | (2) | (3) | (4) |
| --- | --- | --- | --- | --- |
|  | coefficients | | marginal effects | |
|  | medical | travel | medical | travel |
| female | -1.46 | -1.051 | -0.51 | -0.629 |
|  | (28.10) | (7.02) | (9.81) | (4.20) |
| age | -0.888 | -0.452 | -0.31 | -0.27 |
|  | (1.14) | (0.27) | (0.40) | (0.16) |
| married | 32.438 | 24.278*** | 11.282 | 14.434*** |
|  | (29.03) | (7.34) | (10.05) | (4.28) |
| secondary education | 10.263 | 14.417 | 3.598 | 8.728 |
|  | (39.46) | (9.51) | (13.89) | (5.84) |
| higher education | 122.298* | 58.187*** | 44.672* | 36.485*** |
|  | (66.55) | (14.16) | (25.36) | (8.97) |
| income | 97.267*** | 19.575*** | 33.968*** | 11.713*** |
|  | (21.40) | (5.41) | (7.49) | (3.13) |
| municipal area | 46.552* | -23.914*** | 16.311* | -14.247*** |
|  | (28.01) | (8.03) | (9.84) | (4.65) |
| north | -70.338** | 11.943 | -24.058** | 7.201 |
|  | (35.54) | (8.74) | (11.91) | (5.27) |
| northeast | -4.829 | 31.472*** | -1.684 | 19.112*** |
|  | (36.18) | (9.12) | (12.61) | (5.53) |
| south | 48.222 | 63.093*** | 17.136 | 39.650*** |
|  | (33.56) | (17.11) | (12.12) | (10.69) |
| CSMBS | -393.967*** | 14.406 | -115.672*** | 8.737 |
|  | (66.27) | (18.71) | (16.68) | (11.55) |
| SSS | -192.475*** | -21.122 | -62.805*** | -12.429* |
|  | (64.44) | (13.10) | (19.62) | (7.55) |
| dual cover | 89.487 | 73.646** | 32.489 | 47.172** |
|  | (64.83) | (34.18) | (24.46) | (22.63) |
| opt out | 1209.899*** | -135.820*** | 424.867*** | -80.508*** |
|  | (87.53) | (10.44) | (28.88) | (4.99) |
| chronic illness | 234.049*** | 77.069*** | 82.493*** | 46.130*** |
|  | (38.97) | (8.65) | (13.72) | (4.84) |
| year 2021 | -68.707** | -14.679** | -23.924** | -8.772** |
|  | (32.85) | (7.12) | (11.38) | (4.24) |
| CSMBS*year 2021 | 108.858 | 79.957 | 39.951 | 51.647 |
|  | (78.77) | (52.18) | (30.31) | (35.40) |
| SSS*year 2021 | 26.961 | -4.569 | 9.525 | -2.722 |
|  | (81.19) | (16.88) | (29.02) | (10.01) |
| constant | -1869.473*** | -84.094 |  |  |
|  | (250.05) | (59.03) |  |  |
| sigma | 842.644*** | 280.936*** |  |  |
|  | (54.42) | (22.68) |  |  |
| n | 12,014 | 12,014 | 12,014 | 12,014 |
| *Note*. * p<0.10, ** p<0.05, *** p<0.01 | |  |  |  |

Table A4 Two-part model for out-of-pocket health expenditures (inpatient services)

**

**References**

1. Viriyathorn S, Wanwong Y, Rueangsom P, Wangbunjongkun W, Sinam P. Thailand UHC & Overview of the Universal Coverage Scheme of the National Health Security Office. 2020. Available from: <https://eng.nhso.go.th/assets/portals/1/files/01%20UCS%20OVERVIEW%20of%20UHC%20and%20UCS.pdf>.

2. National Health Security Office. NHSO Annual report: Fiscal Year 2021. 2022. Available from: <https://eng.nhso.go.th/assets/portals/1/files/nhso%20report%20fiscal%20year%202022%20-%20Copy.pdf>.

3. Patcharanarumol W, Panichkriangkrai W, Sommanuttaweechai A, Hanson K, Wanwong Y, Tangcharoensathien V. Strategic purchasing and health system efficiency: A comparison of two financing schemes in Thailand. PLOS ONE. 2018;13(4):e0195179. <https://doi.org/10.1371/journal.pone.0195179>

4. Satchanawakul N. Universal Coverage for Emergency Patients (UCEP) in Thailand. 2021. Available from: <https://eng.nhso.go.th/assets/portals/1/files/Thailand_UHC/64-4_UCEP_Book(Eng).pdf>.
